# Exploring Active Case Detection Approaches for Leprosy Diagnosis in Varied Endemic Settings: A Comprehensive Scoping Review

**DOI:** 10.3390/life14080937

**Published:** 2024-07-26

**Authors:** Heynes Brown, Anil Fastenau, Srilekha Penna, Paul Saunderson, Gonnie Klabbers

**Affiliations:** 1 School of Medicine, Dentistry & Biomedical Sciences, Queen’s University Belfast, Belfast BT7 1NN, UK; 2 Department of Health Ethics and Society, Faculty of Health, Medicine and Life Sciences, Maastricht University, Maastricht 6211 LK, The Netherlands; 3 German Leprosy and Tuberculosis Relief Association (GLRA/DAHW), Wuerzburg 97080, Germany; 4 Marie Adelaide Leprosy Centre (MALC), Karachi 74400, Pakistan; 5 Heidelberg Institute of Global Health, University of Heidelberg, Heidelberg 69117, Germany; 6 Department of Global Health, Institute of Public Health and Nursing Research, University of Bremen, Bremen 28359, Germany; 7 American Leprosy Missions, Greenville, SC 29601, USA

**Keywords:** leprosy, active case detection, active case finding, endemicity, early diagnosis

## Abstract

(1) Background: The global burden of leprosy is not shared equally; with the majority of cases being diagnosed in Brazil, India, and Indonesia. Understanding the methods of active case detection (ACD) used in high and low endemic regions is vital for the development of future screening programs. (2) Methods: A systematic search of three databases, PubMed, Embase and Web of Science, was conducted for English language papers, published since the year 2000, which discussed the use of active case detection methods for leprosy screening. The paper utilised the Integrated Screening Action Model (I-SAM) as a tool for the analysis of these methods. (3) Results: 23 papers were identified from 11 different countries. The papers identified 6 different methods of active case detection: Household contact/social contact identification; door-to-door case detection; screening questionnaire distribution; rapid village surveys; school-based screening; and prison-based screening. 15 were located in high endemic regions and 8 of these were located in low endemic regions. (4) Conclusions: For selecting the appropriate methods of active case finding, the leprosy endemicity must be taken into consideration. The findings contribute to policy decision making allowing for more successful future leprosy case detection programs to be designed, ultimately reducing the global burden of the disease, and achieving the WHO’s aim of zero leprosy.

## 1. Introduction

Early case detection is vital for the effective treatment of leprosy, a chronic infectious disease which is caused by infection from the bacteria *Mycobacterium leprae* (*M. leprae*). Successful treatment, without permanent morbidity, is attainable when early and effective case detection has been obtained which allows for swift diagnosis to be achieved [1]. The condition affects the skin and peripheral nervous system, with the World Health Organisation (WHO) classifying the condition as a neglected tropical disease (NTD) as the condition remains a cause of preventable disability for many worldwide [1]. Despite the WHO’s pledge to eradicate the condition in 1990 [2], the condition still affects many across the globe with over 200,000 new clinical diagnoses being made each year in over 120 countries [3]. The disease burden of this condition across the globe is not shared equally however, with only a few countries accounting for the majority of cases worldwide; in 2022 India reported over 103,000 new cases of the infectious disease with Brazil and Indonesia reported over 19,000 and 12,000 new cases respectively within the same year [4]. This disparity in case numbers is reinforced as 150 countries reported fewer than 1000 cases in the same period [4].

In both paucibacillary (PB) leprosy and multibacillary (MB) leprosy, infection will result in the destruction of the peripheral nerves which leads to severe physical disfigurement if not treated effectively [5]. If untreated, the symptoms progress, resulting in nerve damage, often with secondary infections, following trauma, which occurs as a result of peripheral sensation loss [5]. This can lead to loss of cartilage, as well as bone resorption causing nasal collapse and auto-amputation of a patient’s fingers and toes [5]. Due to the severe and appearance-altering impact of severe leprosy, the condition can have an excessive impact on an individual’s mental health. Leprosy diagnosis and disease symptomatology have been associated with an increase in anxiety, depression and suicide among patients [6]. This reduction in mental health status is attributable to many factors including, fear of disease sequelae and fear of death from the condition [6]. Shame further contributes to this decline; with shame and embarrassment being caused by widespread stigmatisation of the disease [6]. This stigma and resulting discrimination take many forms in societies affected by leprosy; unemployment, and marital and social restrictions are common among those diagnosed, and due to the stigma behind the physical deformities associated with the condition, many patients experience avoidance which can result in social isolation [6].

Due to the social and emotional stigmatisation of the condition, described above, and the combined impact of the disease mainly affecting low- and middle-income countries, it is vital to understand the factors contributing to barriers to screening program access. Robb [7] proposed a framework known as the Integrated Screening Action Model (I-SAM) to identify these barriers. It aims to improve understanding of the barriers experienced when accessing screening programs. The I-SAM model can be seen in Figure S1. and is particularly pertinent to discuss when examining leprosy case detection methods because, as shown in the model, the social determinants of health play a vital role in impacting the participant and environmental influences for screening behaviour change [7].

The current literature surrounding the topic of leprosy has yet to discuss which active case detection (ACD) methods are most commonly used in areas of high and low endemicity. Moreover, it is yet to be reviewed how these methods act to remove the barriers to screening faced by local populations across the globe. This paper aims to address the research question: ‘What are the different active case detection methods being used for early case detection of leprosy in high and low endemic regions?’ The objectives of the paper include: To review the current literature to gain an understanding of the different methods of active leprosy case detection used across the globe; to understand which leprosy active case detection methods are used in high endemic regions and in low endemic regions; and to gain an understanding of how these methods alleviate the barriers to leprosy diagnosis in high and low endemic regions. The findings of this study will allow recommendations to be drawn, informing policymakers designing intervention implementation on which programs are best suited depending on the disease endemicity and barriers to screening faced by the local population. It is expected that the results of this review will enable more effective and efficient case detection of leprosy in high and low endemic areas and therefore an improvement in early case detection will be achieved; reducing disease burden and improving quality of life for the hundreds of thousands newly diagnosed with leprosy annually.

## 2. Materials and Methods

### 2.1. Design

The scoping review was carried out according to the Preferred Reporting Items for Systematic Reviews and Meta-Analyses (PRISMA) guidelines for scoping reviews. The PRISMA checklist can be seen in Table S1.

### 2.2. Eligibility Criteria

The eligibility criteria for this review were defined as the following: English language papers from the years 2000 to 2023 inclusive. Furthermore, papers published detailing active case detection in any country were included in the review. Further inclusion criteria were free full-text availability, and primary peer-reviewed research, with clear specifications on the active case detection method used for leprosy detection.

### 2.3. Search Strategy

The search strategy was conducted using three databases, namely, PubMed, Embase and Web of Science. Controlled vocabulary as well as free search terms were used to build the search string, which is presented in Table S2. The date of the most recent search was the 31 May 2023. All search results were exported into EndNote 20.3 for processing and screening, with Rayyan software being used for further deduplication and processing.

### 2.4. Data Extraction

Data were extracted on: the location of the study, the type of case detection method used; the methods of diagnostic procedure used; the endemicity of the study location with respect to leprosy; and setting of the study location, be that community, hospital, school or prison. Screening program population size was also extracted, alongside, the number of new leprosy cases detected by the studies. Regarding the extraction of endemicity, this was either provided by the study and was recorded as high or low accordingly. When endemicity was not explicitly mentioned in the study, it was calculated by applying the new case detection rate (NCDR) prior to ACD implementation in the region. This figure must have been detailed in the study and high endemicity was defined as regions with greater than or equal to 1 new case detected per 10,000 population, with low endemicity being defined as those with less than 1 new case detected per 10,000 population. If new case detection rate was not documented in the study, the prevalence rate (PR) of leprosy within the region was used to calculate endemicity. A PR of less than 1 per 10,000 was considered to be a region of low endemicity and a region with greater or equal to 1 per 10,000 prevalence was equated to a region of high endemicity [8].

### 2.5. Data Extraction Table

Table S3 shows the data extracted from the papers identified which were included in the review.

### 2.6. Data Analysis

The data extracted were analysed based on the method of ACD used, the endemicity of the study location, and the number of new cases detected per screening program. This included analysis of the frequency, distribution and average percentage of cases detected per ACD method used in high and low endemic areas.

### 2.7. Ethical Considerations

Ethical Consideration was submitted and approved by Maastricht University.

## 3. Results

**Figure 1:**
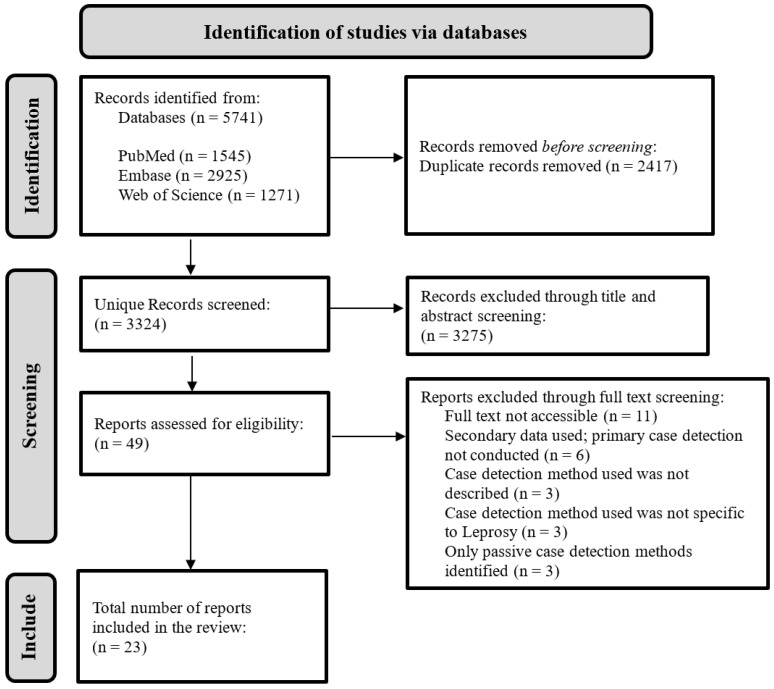
Flowchart of the database’s search results inclusion and exclusion criteria.

The systematic search yielded a total of 5741 results. After de-duplication and screening, 23 papers were included. This screening process is shown in Figure 1. The majority of papers were excluded through title and abstract screening. Papers were excluded if they did not detail one, or more, method(s) of active case detection used for identifying leprosy cases within a specific population.

All studies included in the review mentioned forms of ACD for leprosy and these studies were located in 11 different countries. The locations with the highest frequency of studies included in the review were India and Brazil. 7 included details of ACD campaigns located in Brazil [9,10,11,12,13,14,15] and 6 studies included details of ACD methods conducted in India [16,17,18,19,20,21]. 2 studies detailed ACD campaigns in China [22,23]. Details of ACD campaigns in Bangladesh [24], Cambodia [25], Colombia [26], Comoros [27], Indonesia [28], Malaysia [29], Nepal [30] and Sri Lanka [31] were also included in the review as each country was represented in one distinct paper. No studies from high income countries were identified by the review. 17 of the studies detailed community based ACD campaigns [9,10,11,12,13,15,16,17,18,19,20,21,22,23,24,26,27,28,29]. 2 studies conducted case detection campaigns within schools, focusing on schoolchildren [14,19]. 2 studies also gave details of ACD programs occurring within prison populations [12,30]. 3 studies were conducted away from a community setting, with these studies using hospital records to identify index cases from which ACD could be based [23,25,31].

The ACD methods identified fell into 6 categories: Household contact (HHC) or social contact (SC) identification; door-to-door case detection (D2D); screening questionnaire distribution (SQ); rapid village surveys (RVS); school-based screening (SS); and prison-based screening (PS). Some of the studies consisted of a mixture of these ACD methods: 11 studies included the use of household or social contact identification [10,13,14,23,24,25,26,27,28,30,31]; 10 studies mentioned the use of door-to-door screening methods [10,11,13,16,17,18,20,21,29,30]; 5 studies detailed use of screening questionnaire distribution [9,12,13,17,18]; 2 studies stated the use of a rapid village survey [15,22]; 1 study used school-based screening [19]; and 1 study used prison-based screening for active detection of leprosy cases [30]. 21 of the 23 studies used physical medical examination (PME) as a diagnostic procedure for confirmation of leprosy diagnosis [9,10,11,12,13,15,16,17,18,19,20,21,22,23,24,25,26,27,29,30,31]. 12 studies used laboratory testing alongside PME for diagnosis of leprosy [9,10,12,13,15,16,17,20,23,26,29,31] and 2 studies solely utilised laboratory testing, for diagnostic analysis [14,28].

Only 10 papers mentioned the specific endemicity level of the study location [9,10,11,14,15,23,25,27,28,29]. 1 paper did not disclose either of these endemicity measurements [19], and therefore endemicity was regarded as high or low depending on state figures for the study location during the same time period that the study was conducted. Overall, it was found that 15 studies were located in high endemic regions [10,11,12,13,14,16,19,20,21,26,27,28,29,30,31] and that 8 studies were located in low endemic regions [9,15,17,18,22,23,24,25].

### 3.1. Diagnostic Procedures

These procedures were used alongside the ACD methods to formally diagnose patients suspected of being infected with *M. leprae*. These diagnostic procedures are categorised into PME and laboratory testing. 21 studies included the use of PME [9,10,11,12,13,15,16,17,18,19,20,21,22,23,24,25,26,27,29,30,31]. This consisted of a dermato-neurologic examination. Some studies also included the use of Semmes-Weinstein monofilaments for examination of light sensation ability [10,13,16]. 14 studies utilised laboratory testing when diagnosing leprosy patients. These tests consisted of slit-skin smear testing [16,17,20,23,29,31], skin biopsy examination [16,23,29], anti- phenolic glycolipid-I (PGL-I) and/or anti-Leprosy Infectious Disease Research Institute (IDRI) Diagnostic-1 (LID-1) serology testing [9,12,15,26] and polymerase chain reaction (PCR) analysis [9,14,23,26,28]. Peripheral nerve ultrasonography [12] was used as well as the lepromin test [26] for diagnosis of leprosy amongst suspected individuals.

### 3.2. Case Detection Methods Identified

#### 3.2.1. Household Contact or Social Contact Identification

10 studies looked at HHC transmission [10,13,23,24,25,26,27,28,30,31] and these studies all had similar definitions of household contact, with one study defining household contact as, “persons who live together with the index case or frequent visitors to the household” [31] household was defined as, “a group of people sharing a roof and/or kitchen in their usual residence” [24]. Social contacts include, neighbourhood contacts, defined in one study as “living within a radius of about 200 metres around the index patient,” [23] and school contacts, which were defined as, “(a) participant under the age of 15 who stayed in a school environment for an average of 4 h a day with confirmed cases of leprosy reported” [14]. 7 studies investigated neighbourhood transmission, through ACD targeted at neighbourhood contacts, alongside HHC transmission [10,13,23,25,27,28,29,30]. Whereas one study focused distinctly on school contact transmission, choosing to undertake ACD amongst the school contact population [14]. Index patients were identified using different methodologies depending on the study. Some studies identified index patients using hospital records of current and past patients [23,25,31], whereas others used home visits to neighbourhoods to identify index patients [10,13,30], and finally one identified social contact through school contact investigation [14].

#### 3.2.2. Door-to-Door Detection

Door-to-door detection, otherwise known as, house-to-house detection in some papers reviewed, is the methodical and targeted assessment of all individuals identified in a particular community of suspects. This ACD method utilised home visits for individuals who fit the participant inclusion criteria for the study. In the papers identified, these included the screening of specifically identified individuals, in the case of household or social contact identification [10,13,30], or more generalised individuals, in the case of village wide screening programs [11,16,17,18,20,21,29]. D2D screening programs utilised ACD teams. The composition of the ACDs varied from study to study, however, many of the studies reviewed, included volunteer health workers who would travel from door-to-door interviewing patients. The volunteers would then distribute a screening questionnaire, or, alongside a trained medical professional, undertake a PME as part of the screening and diagnostic process of leprosy.

#### 3.2.3. Screening Questionnaire Distribution

**Figure 2:**
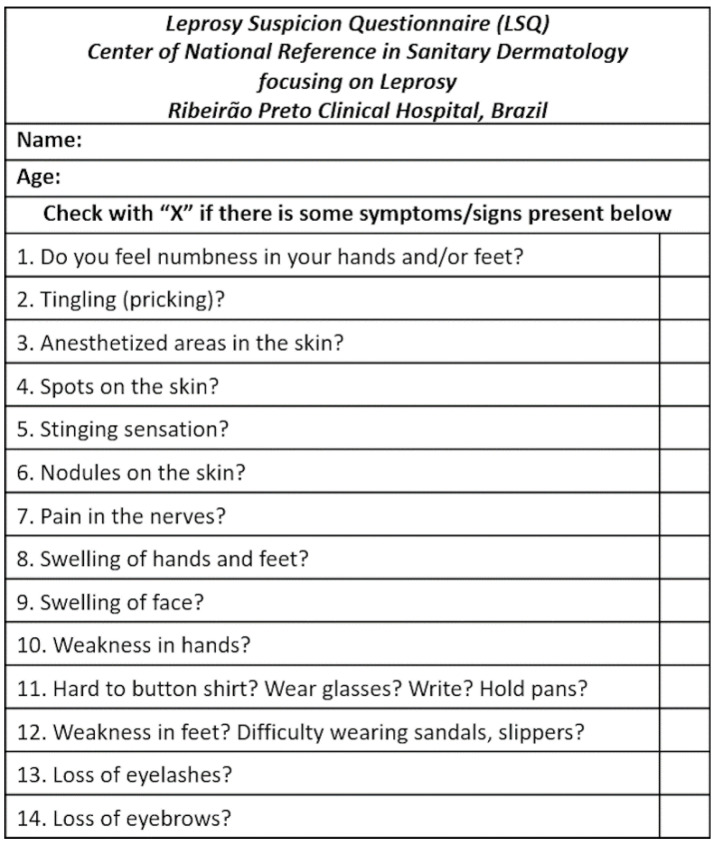
An example of a LSQ distributed as part of a SQ ACD method (Reprinted from Ref. [9]).

It was found that 5 studies detailed the use of a questionnaire that screening program participants completed as part of the case detection process [9,12,13,17,18]. This questionnaire was used as part of the identification process of individuals who may be displaying signs and symptoms of leprosy, and these patients were then invited to undertake either physical or laboratory diagnostic procedures to confirm the differential diagnosis of leprosy depending on the answers given to the questionnaire. The questionnaires were referred to as Leprosy Suspicion Questionnaires (LSQ) in two of the studies identified [9,12]. These questionnaires asked the patient about the expression of any classical signs or symptoms of leprosy and whether they had been in contact with anybody who had been diagnosed with the condition previously. Most interestingly, one of the LSQs, seen in Figure 2, enquired about the development of neurological symptoms [9]. The inclusion of neurological specific questioning is a novel approach to SQ distribution and allows for clear identification of cases where neuropathy has developed as a result of leprosy infection. This method of case detection does, however, require a certain level of patient literacy to be effective. Other questionnaires included the use of educational flashcards to which the patient could identify the signs described and direct the researcher to any family members who may be expressing these classical symptoms [18]. These pictorial SQ distribution methods reduced the need for high levels of participant literacy, however, it is important to note that it is difficult to capture neurological symptom burden when using this approach. Questionnaires also contained questions relating to social and environmental aspects of life, which enabled social contacts to be identified if needed [13]. All of these studies were carried out in a community setting and one of the papers identified, utilised a LSQ when screening for leprosy patients within a female prison population [12].

#### 3.2.4. Rapid Village Survey

Two studies documented the use of a rapid village survey [15,22]. One documented the use in 91 villages where community leaders and medical professionals, documented in the study as the ‘Survey Team’, carried out the RVS [22]. The process occurred as follows: Prior to the commencement of the survey, the local village populations were primed to self-screen for any signs or symptoms of leprosy. This was accomplished as pictorial posters were distributed to local medical professionals in the target areas which included images of classical leprosy signs. The survey team remained in the area for up to one day and during this time, free leprosy screening was provided using PME by medical professionals after which, any suspected patients, i.e., those who displayed classical signs of the disease, were offered laboratory diagnostic testing. During the screening process, any close household contacts of suspected patients were identified, and contact screened. Education for teachers, community leaders and rural doctors, on the importance of regular screening, was further facilitated through group discussions [22]. The other paper documented the use of a mobile clinic to conduct the screening which had the ability to undertake physical medical examination and laboratory testing for leprosy diagnosis [15]. The clinic was stationed at the major bus terminal in the study location, a populous area within the city. The mobile clinic was advertised prior to the arrival, using forms of media such as radio, television and banner advertisements in the city. Patients were also asked to inform staff if they were, or previously had been close relatives or social contacts of positive leprosy patients [15].

#### 3.2.5. School-Based Screening

One study conducted a school-based screening program for leprosy cases [19]. Here, 26 high schools from a mixture of urban and rural locations were selected to be a part of the study. 5–15 ‘student leaders’ were chosen from each school. These were participants who would represent the screening program amongst their peers. These volunteers, along with the schools’ teachers, would participate in an education session relating to leprosy signs and symptoms. They were also taught basic examination technique for these signs. Those who attended the education session were encouraged to pass any knowledge learnt on to their wider classmates and students. After 2 weeks a list of suspects was collated by those who attended the education lesson, and a trained leprosy supervisor was invited to screen suspected leprosy cases within the schools. Any suspected cases after this were invited to attend a PME at a local hospital for confirmation of diagnosis.

#### 3.2.6. Prison-Based Screening

Two papers documented the use of ACD methods within prison populations [12,30], however, one focused on the use of LSQs has been documented accordingly [12]. In the other paper, the ACD method described was the use of prison populations as sites of convenience sampling [12]. In the study, 6 different prison populations were sampled and a total of 4428 prisoners were screened using this method of case detection. Diagnosis was confirmed using PME.

### 3.3. Endemicity

#### 3.3.1. High Endemic Regions

As described above, 15 of the 23 studies identified were located in high endemic regions [12,30]. Of these, only 6 studies explicitly mentioned endemicity [10,11,14,27,28,29]. The remaining were categorised depending on NCDR or PR. 4 of the remaining studies detailed the NCDR to be greater than or equal to 1 per 10,000 [16,20,21,31] and 4 studies detailed the PR within the region to be greater than 1 per 10,000 [12,13,26,30] categorising the studies as being from high endemic regions. The study which failed to mention leprosy endemicity was located in Tamil Nadu, India [19]. This study was reported to be in an area regarded as high endemic for leprosy in the year the study was conducted [32]. Regarding the location of the high endemic studies. Out of all the studies located in high endemic regions, Brazil had the highest frequency of papers included in the review, with 5 studies detailing ACD methods located in high endemic regions of the country [10,11,12,13,14]. India had 4 studies located in high endemic regions [16,19,20,21], with Sri Lanka [31], Malaysia [29], Nepal [30], Comoros [27], Indonesia [28] and Columbia [26] all having one study located in a high endemic region within the country.

**Figure 3:**
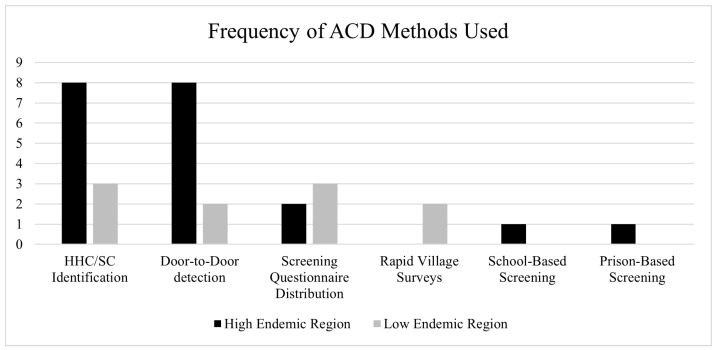
Frequency of identification of ACD methods used in high and low endemic regions.

Figure 3 shows the frequency of occurrence of the different methods of ACD in high and low endemic areas. In high endemic regions 3 papers detailed use of more than one method of ACD within the single ACD campaign [10,13,30].

#### 3.3.2. Low Endemic Regions

Regarding studies located in low endemic regions, 4 of the 8 papers mentioned the endemicity of the region explicitly [9,15,23,25]. One study recorded the prevalence of leprosy using NCDR. This was recorded to be an average of 0.43 per 10,000, below the threshold of 1 per 10,000 [17]. 3 papers used prevalence as a measurement of endemicity: all stating it to be below 1 per 10,000 [18,22,24].

With respect to the location of the low endemic studies identified: Brazil [9,15], China [22,23] and India [17,18] all had the highest frequency of low endemic studies identified; with each country being mentioned in 2 low endemic studies respectively. One low endemic study was recorded in both Bangladesh [24] and Cambodia [25].

#### 3.3.3. Comparison between High and Low Endemic Regions

**Figure 4:**
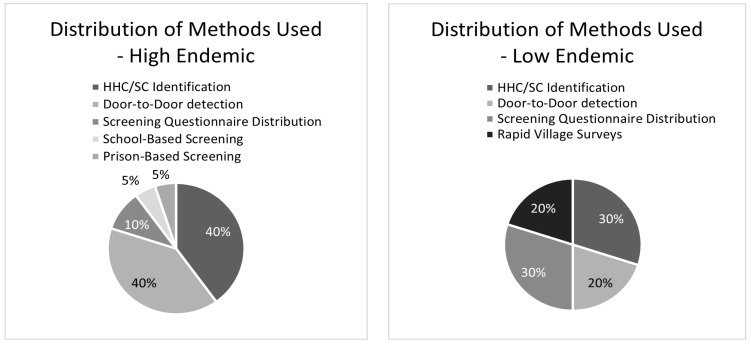
Distribution of ACD methods used in high and low endemic regions.

Figure 3 shows a comparative distribution of the ACD methods for case detection in the high and low endemic areas identified. Figure 4 shows the proportionality of the frequency of these case detection methods used in high and low endemic regions.

### 3.4. Effectiveness of the Active Case Detection Methods

When studying the effectiveness of case detection campaigns, two metrics are important, firstly the total number of participants screened and secondly the case detection rate. Table 1 shows the average amount of people screened per case detection method in each respective endemic category. Here we are able to see that on average, more were screened in low endemic areas compared to high endemic areas.

**Figure 5:**
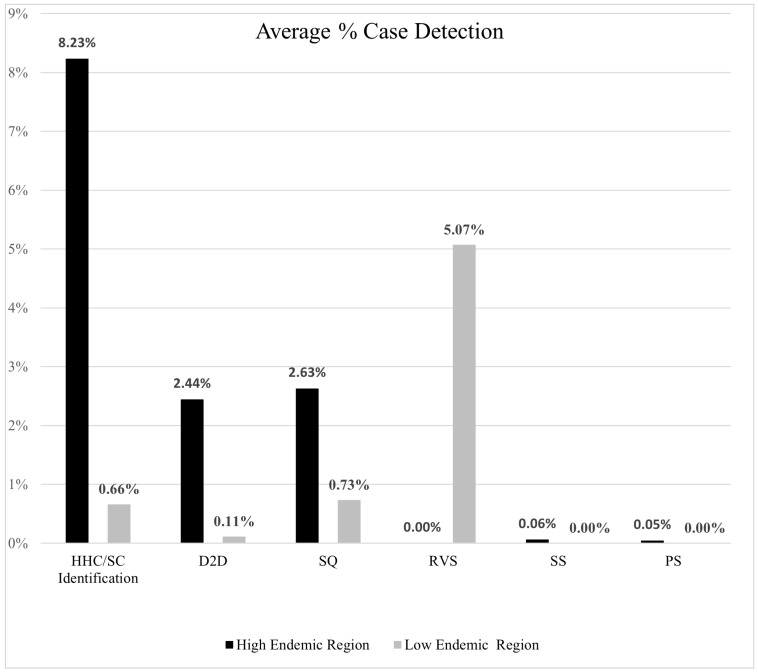
Average percentage of cases detected per ACD method in high and low endemic settings.

Regarding the average case detection rate of each method of ACD in high and low endemic regions, more cases were detected in high endemic areas compared to low endemic areas. This can be seen in Figure 5.

## 4. Discussion

In this study, we investigated the methods of active case detection used in high and low endemic regions. We screened 5741 results from three major scientific journal databases, identifying 23 relevant papers from 11 countries. Six ACD methods were identified: Household contact or social contact identification, door-to-door case detection, screening questionnaire distribution, rapid village surveys, school-based screening, and prison-based screening.

In high endemic regions, 15 studies employed a wider range of ACD methods, including HHC/SC Identification, D2D case detection, SQ distribution, SS, and PS. In contrast, low endemic regions mainly used HHC/SC identification, D2D case detection, SQ distribution, and RVS. SS and PS were exclusively used in high endemic regions, while RVS was solely used in low endemic regions.

This review reveals a global diversity in ACD methods for identifying and diagnosing leprosy cases. Figure 4 demonstrates how ACD screening programs differ based on endemicity. High-endemic areas predominantly utilised HHC/SC identification and D2D case detection, while low endemic regions employed a more evenly distributed range of methods. Additionally, it was observed that more individuals were screened per detection campaign in low endemic regions compared to high endemic regions in the 3 commonly shared comparable campaigns of HHC/SC identification, D2D screening and SQ distribution. However, the latter exhibited a higher rate of case detection, as illustrated in Figure 5.

The findings, detailed in Figure 5, indicate that in high endemic regions, HHC/SC identification methods of ACD are most successful at identifying cases, with the average case detection percentage of this method being 8.23%. When observing case detection in low endemic regions, Figure 5 indicates that RVSs are most successful at detecting leprosy cases with the average case detection percentage being 5.07%. Moreover, when comparing case detection using the comparable methods of HHC/SC distribution, D2D and SQ distribution, Figure 5 indicates that using these methods in high endemic regions yields a greater number of percentage cases detected. It is also seen that SS and PS in high endemic regions yields a relatively small percentage case detection of 0.06% and 0.05% respectively.

Furthermore, it is important to screen as many individuals as possible when conducting an ACD campaign for leprosy. This is particularly true within a low endemic region, due to the widely distributed nature of cases. Thus, an active case detection method which is able to screen large quantities of individuals is preferable. Using Table 1, we can see that D2D and SQ distribution were most effective at screening a large number of individuals, with the average number of people screened per intervention being 515,325 and 344,630 respectively.

The variability in effectiveness of the different methods of ACD observed between regions of similar endemicity, may be explainable using the I-SAM model. The model suggests that a participant motivation, capability, or environmental opportunity, combine to alter the attendance of screening programs by local populations. The success of certain campaigns over others may stem from their ability to influence screening behaviour, by altering these different factors, to encourage individuals to engage in proactive leprosy screening through PME and laboratory testing. Each ACD method uniquely influenced participant and environmental factors, facilitating a shift in screening behaviour, however, some had a larger impact than others.

Current literature on leprosy case delays emphasises the impact of participant and environmental factors on hindering prompt detection globally. Dharmawan et al.’s [33] systematic review underscores reduced health-service-seeking behaviour, where patients often resort to traditional or alternative medicine providers as initial care, leading to delays in case detection. For example, one study revealed that 70% of leprosy patients did not seek medical advice despite observing symptoms, indicating issues with patient motivation, capability, and environmental opportunities for accessing screening programs. Henry et al.’s [34] research highlighted reduced health literacy and increased social stigma as key factors contributing to patient delay and aligns with the I-SAM model, emphasising the impact of motivation and capability on healthcare-seeking behaviour. Patients’ limited perception of risk and medical literacy hindered their understanding and recognition of symptoms, reducing their engagement with screening programs. Further, Henry et al. underscored how social stigmatisation leads to delayed leprosy case detection, causing participants to fear community isolation even if they understood their symptoms [34]. This fear, stemming from societal stigma, influences screening behaviour, as outlined in the I-SAM model. Additionally, Dharmawan et al. highlighted physical barriers, including financial, logistical, and staffing issues, as well as geographical circumstances, leading to delays in leprosy case detection [35]. These findings illustrate how both social and physical environmental factors significantly impact engagement in leprosy screening programs. The intersection of participant and environmental influences underscores the need for effective active case detection methods to address these delays.

Using the I-SAM model as a lens through which to view these methods of ACD, it is clear to see the benefits that these types of case detection methods bring to both high and low endemic regions. Firstly, HHC/SC identification mostly influenced the participant influences identified by the I-SAM model which allowed for a change in participant screening behaviour to occur. This method of ACD mostly influences the subcategory of ‘motivation’ related participant influences affecting action. With this method, the participants of the study are notified of their increased relative risk of infection through being close household or social contacts of a previously diagnosed leprosy patient. Through the explicit and inferred increase in their risk status, the participants’ perceived personal risk is heightened, and this changes the emotional influence of the situation, fear, ultimately altering the balance of benefits and harms within the individual’s mind causing a change in screening behaviour to occur [7].

Moreover, the process of D2D case detection has an influence on the participant and environmental influences, described by the I-SAM model, which oftentimes hinders screening program attendance. This method altered the ‘capability’ element of participant influences as well as the ‘opportunity’ element of environmental influences. This case detection method specifically changes the physical component of environmental influence. D2D case detection removes the barriers of financial expense for travel, for example, as well as removing other hindering factors such as disability associated barriers which may cause screening delays. By removing these factors, through D2D case detection, the physical environmental influences can be changed or removed to allow for an improved access to screening. For example, the patient’s physical access to healthcare is improved and any physical barriers to accession of screening are removed such as health service organisation as described by Dharmawan et al. [35]. By eliminating these factors through increasing screening convenience, D2D screening allows for an increase in uptake of screening programs as seen by the magnitude of participants screened in the papers included in this review.

Furthermore, regarding SQ distribution, this method of ACD mostly changes the participant influences which have been hindering screening program attendance. This method of case detection influences the ‘motivation’ and ‘capability’ elements of participant influences shown in the I-SAM model. SQ distribution changes the ‘capability’ for screening as the participant is made more aware of the signs and symptoms, either through the questions enclosed within the questionnaire or through the pictures contained in the flashcards. This therefore influences the participant’s ‘motivation’ as they ultimately have a wider knowledge regarding the risk of their infection status from *M. leprae* and the participant’s perceived risk thus changes, forcing them to act positively towards leprosy screening.

RVSs act in a slightly different manner when changing screening behaviour. As RVS include an educational awareness campaign either concurrently or prior to the arrival of the screening element. This improves the ‘capability’ participant influence described by the I-SAM model. This is achieved as the participants’ knowledge of signs or symptoms increases, allowing self-screening to occur before arriving for medical screening as part of the formal RVS. This also serves to increase the participants’ ‘motivation’ influence as by becoming more educated on the signs and symptoms of leprosy the participant will have a greater understanding of their perceived risk of infection. Moreover, RVS improve the social and physical ‘opportunity’ environmental influence of screening program attendance; RVS provide community endorsement for attending the screening program. This takes the form of mass media advertising of the program prior to the arrival, alongside endorsements from local healthcare professionals. Moreover, regarding the change of the physical environmental influences, as RVSs are located in convenient areas for the entire village they remove any potential barriers to access for the local community. The combination of the change in these environmental factors allows for the community to safely partake in leprosy screening, improving engagement with such programs.

SS and PS programs resemble D2D programs but are tailored for specific environments, such as schools or prisons. Norman et al.‘s SS initiative focused on enhancing participant awareness within the school community, thereby improving both participant capability and motivation for change through education on symptoms and facilitating self-screening. By leveraging school visits, the program enhanced the physical environmental opportunity, ensuring easier access to the screening program. The authors highlighted the importance of school screening as school children are an “easily accessible target group” and that “enhancing awareness among children could… have a ‘ripple effect’ amongst their families and communities” [19]. This highlights the importance of school screening and the potential benefits of conducting screening programs amongst this age group. Moreover, with respect to PS, the authors describe the importance of screening such a specific population. The authors describe the increased risk that prisoners have regarding leprosy transmission; this is due to the cramped environment and living conditions alongside poor diet and lack of personal hygiene [30]. Therefore, it has been identified that prison populations are at higher relative risk of leprosy transmission compared with the general population [30]. Moreover, as prison inhabitants are restricted to a single environment, their ability to attend other screening programs is much more reduced, and thus, improving the physical environmental ‘opportunity’ for screening is imperative; something which is achieved through PS.

The mentioned case detection methods effectively combat the stigma surrounding leprosy, countering negative screening experiences linked to fear and fatalistic views. By removing the burden of attending screenings from participants, these methods facilitate easier engagement. Examining these methods through the lens of the I-SAM model reveals their ability to modify participant and environmental influences, thereby improving motivation, capability, and environmental opportunities for positive screening behaviour. Consequently, these measures contribute to the increased diagnosis and treatment of leprosy cases.

No sources identified in this review included details of skin NTD screening via the novel skin camp approach. These community-based interventions have been proposed as an effective method of screening as they are implemented at a community level, in close partnership with the local community leaders and organisations. Moreover, these interventions are extremely effective at reducing the stigma associated with leprosy screening program attendance as individual disease disclosure is not necessary and using an integrated approach, more than one skin condition is screened for at any one time [36].

### 4.1. Strengths and Limitations

The review adhered to the PRISMA guidelines, ensuring methodological reliability. However, limitations included the exclusion of studies from all leprosy-endemic countries due to language constraints, limited database searches, and paper inaccessibility. This hindered a comprehensive understanding of global ACD campaigns. Additionally, the study’s reliance on the I-SAM model didn’t capture the lived experiences of participants, restricting insights into the qualitative reasons behind the (un)successful nature of ACD programs and barriers to screening.

### 4.2. Recommendations for Future Research and Practice

Based on the research findings from this review it is recommended that policymakers implement case detection campaigns using methods of ACD which influence the participant’s participant and environmental influences for change together. This may consist of utilising more than one method of ACD in the same campaign, for example, the use of HHC/SC identification alongside D2D screening or through a single campaign such as RVS. By implementing ACD campaigns that focus on jointly influencing the participant and environmental influences for change, it is hoped that a greater breakdown of barriers to screening will be achieved, allowing for a more effective ACD campaign. Moreover, it is vital to understand the lived experience of those who have undergone these programs, in order to provide maximum efficiency when conducting future ACD campaigns. Thus, it is recommended that qualitative research is carried out to understand this further in the context of the participants’ beliefs and opinions regarding leprosy case detection participation. Moreover, as shown in this review, leprosy diagnosis is best achieved when active case detection goes side-by-side with swift and effective diagnosis using efficient and effective diagnostic tools. Therefore, it is recommended that research is conducted into implementing new molecular diagnostics for diagnosing leprosy as described by Mohanty et al. [37] alongside active case detection methods.

It also recommended that more research is carried out into the implementation of SQ distribution in areas of high and low population literacy to identify if the effectiveness of this ACD method is altered when conducting these methods in areas of differing population literacy.

## 5. Conclusions

To conclude, this review set out to identify the different methods of ACD used in high and low endemic areas for leprosy. Firstly, it began by identifying methods of ACD used globally. The findings included: Household contact or social contact identification; door-to-door case detection; screening questionnaire distribution; rapid village surveys; school-based screening; and prison-based screening. By categorising the use of these ACD methods into high and low endemic regions it was clear which methods were used in high and low endemic areas. Household contact or social contact identification, door-to-door case detection and screening questionnaire distribution were all used in both endemic regions; however, rapid village surveys were only used in low endemic regions and school-based screening and prison-based screening were only used in high endemic regions. Using the I-SAM model as a theoretical lens for analysis, it was identified that influencing both the participant and environmental influences for change is vital for successful ACD rollout in order to achieve the WHO’s global goals for leprosy diagnosis and treatment.

## Figures and Tables

**Table 1:** Average amount of participants screened per ACD campaign.

	Average Amount of People Screened—High Endemic	Average Amount of People Screened—Low Endemic
HHC/SC Identification	1314	18,191
D2D	412,426	515,325
SQ	397	344,630
RVS	-	897
SS	23,125	-
PS	4428	-

## Supplementary Materials

The following supporting information can be downloaded at: https://www.mdpi.com/article/10.3390/life14080937/s1, Figure S1: S1_Figure_I-SAM Model; Table S1: S1_PRISMA-ScR-Checklist; Table S2: S2_Search String Table; Table S3: S3_Data Extraction Table.

## References

[1] Hambridge T., Coffeng L.E., de Vlas S.J., Richardus J.H. (2023). Establishing a standard method for analysing case detection delay in leprosy using a Bayesian modelling approach. Infect. Dis. Poverty.

[2] Chen K.H., Lin C.Y., Su S.B., Chen K.T. (2022). Leprosy: A review of epidemiology, clinical diagnosis, and Management. J. Trop. Med..

[3] WHO Leprosy. World Health Organization. https://www.who.int/news-room/fact-sheets/detail/leprosy.

[4] Jonnalagada S. Number of New Leprosy Cases. https://www.who.int/data/gho/data/indicators/indicator-details/GHO/number-of-new-leprosy-cases.

[5] Makhakhe L. (2021). Leprosy review. S. Afr. Fam. Pract..

[6] Somar P., Waltz M., van Brakel W. (2020). The impact of leprosy on the mental wellbeing of leprosy-affected persons and their family members—A systematic review. Glob. Ment. Health.

[7] Robb K.A. (2021). The integrated screening action model (I-SAM): A theory-based approach to inform intervention development. Prev. Med. Rep..

[8] Ogunsumi D.O., Lal V., Puchner K.P., van Brakel W., Schwienhorst-Stich E.-M., Kasang C., Chukwu J., Kreibich S., Parisi S., Richardus J.H. (2021). Measuring endemicity and burden of leprosy across countries and regions: A systematic review and Delphi survey. PLoS Negl. Trop. Dis..

[9] Bernardes Filho F., Silva C.M., Voltan G., Leite M.N., Rezende A.L., de Paula N.A., Barreto J.G., Foss N.T., Frade M.A. (2021). Active search strategies, clinicoimmunobiological determinants and training for implementation research confirm hidden endemic leprosy in inner São Paulo, Brazil. PLoS Negl. Trop. Dis..

[10] Moura M.L., Dupnik K.M., Sampaio G.A., Nobrega P.F., Jeronimo A.K., do Nascimento-Filho J.M., Miranda Dantas R.L., Queiroz J.W., Barbosa J.D., Dias G. (2013). Active surveillance of Hansen’s Disease (leprosy): Importance for case finding among extra-domiciliary contacts. PLoS Negl. Trop. Dis..

[11] Nery J.A.d.C., Pimentel M.I.F., Lyra M.R., Sohsten B.d.L.V., Marinho D.P., Périssé A.R.S. (2012). Detection of clusters of leprosy cases among Guarani Indians in the southern region of the State of Rio de Janeiro, Brazil. Rev. Soc. Bras. Med. Trop..

[12] Silva C.M., Bernardes Filho F., Voltan G., Santana J.M., Leite M.N., Lima F.R., Santana L.D., de Paula N.A., Onofre P.T., Marques-Junior W. (2021). Innovative tracking, active search and followup strategies for new leprosy cases in the female prison population. PLoS Negl. Trop. Dis..

[13] de Campos D.C.C., Dutra A.P.B., Suares V.L., de Carvalho P.A.C., Camargo L.M.A. (2015). New strategies for active finding of leprosy cases in the Amazonian region. Rev. Soc. Bras. Med. Trop..

[14] Sato C.M., Rodrigues T.d.S.V., Silva P.R.d.S., dos Santos E.S., Xavier D.R., Baptista I.M.F.D., Cortela D.d.C.B., Ignotti E., Ferreira S.M.B. (2021). Social school contacts of multibacillary leprosy cases in children living in the hyperendemic region of the Midwest of Brazil. J. Pediatr..

[15] Frade M.A.C., de Paula N.A., Gomes C.M., Vernal S., Filho F.B., Lugão H.B., de Abreu M.M.M., Botini P., Duthie M.S., Spencer J.S. (2017). Unexpectedly high leprosy seroprevalence detected using a random surveillance strategy in midwestern Brazil: A comparison of ELISA and a rapid diagnostic test. PLoS Negl. Trop. Dis..

[16] Shetty V.P., Thakar U.H., D’souza E., Ghate S.D., Arora S., Doshi R.P., Wakade A.V., Thakur D.V. (2009). Detection of previously undetected leprosy cases in a defined rural and urban area of Maharashtra, Western India. Lepr. Rev..

[17] Kumar A., Girdhar A., Chakma J.K., Girdhar B.K. (2013). Detection of previously undetected leprosy cases in Firozabad District (U.P.), India during 2006-2009: A short communication. Lepr. Rev..

[18] Kumar M.S., Padmavathi S., Shivakumar M., Charles U., Appalanaidu M., Perumal R., Thiagarajan P.N., Somasekhar Y. (2015). Hidden leprosy cases in tribal population groups and how to reach them through a collaborative effort. Lepr. Rev..

[19] Norman G., A Joseph G., Udayasuriyan P., Samuel P., Venugopal M. (2004). Leprosy case detection using schoolchildren. Lepr. Rev..

[20] Rao P.V.R., Bhuskade R.A., Desikan K.V. (2000). Modified leprosy elimination campaign (MLEC) for case detection in a remote tribal area in the State of Orissa, India. Lepr. Rev..

[21] Shetty V.P., Pandya S.S., Arora S., Capadia G.D. (2010). Observations from a ‘special selective drive’ conducted under National Leprosy Elimination Programme in Karjat taluka and Gadchiroli district of Maharashtra. Indian J. Lepr..

[22] Chen S., Zheng Y., Zheng M., Wang D. (2007). Rapid survey on case detection of leprosy in a low endemic situation, Zhucheng County, Shandong Province, The People’s Republic of China. Lepr. Rev..

[23] Wang N., Chu T., Li F., Wang Z., Liu D., Chen M., Wang H., Niu G., Liu D., Zhang M. (2020). The role of an active surveillance strategy of targeting household and neighbourhood contacts related to leprosy cases released from treatment in a low-endemic area of China. PLoS Negl. Trop. Dis..

[24] Butlin C.R., Nicholls P., Bowers B., Singh S., Alam K., Quilter E. (2019). Household contact examinations: Outcome of routine surveillance of cohorts in Bangladesh. Lepr. Rev..

[25] Cavaliero A., Ay S.S., Aerts A., Lay S., So V., Robijn J., Steinmann P. (2021). Preventing leprosy with retrospective active case finding combined with single-dose rifampicin for contacts in a low endemic setting: Results of the Leprosy Post-Exposure Prophylaxis program in Cambodia. Acta Trop..

[26] Cardona-Castro N., Beltrán-Alzate J., Manrique-Hernández R. (2008). Survey to identify Mycobacterium leprae-infected household contacts of patients from prevalent regions of leprosy in Colombia. Mem. Do Inst. Oswaldo Cruz.

[27] Ortuño-Gutiérrez N., Mzembaba A., Baco A., Braet S.M., Younoussa A., Salim Z., Amidy M., Grillone S., Said A., de Jong B.C. (2022). High yield of retrospective active case finding for leprosy in Comoros. PLoS Negl. Trop. Dis..

[28] Krismawati H., Oktavian A., Maladan Y., Wahyuni T. (2020). Risk factor for Mycobacterium leprae detection in household contacts with leprosy patients: A study in Papua, East Indonesia. Med. J. Indones..

[29] Utap M.S., Kiyu A. (2017). Active case detection of leprosy among indigenous people in Sarawak, East Malaysia. Lepr. Rev..

[30] Mahato R.K., Ghimire U., Lamsal M., Bajracharya B., Poudel M., Naapit P., Lama K., Dahal G., Hayman D.T., Karna A.K. (2022). Epidemiology of leprosy identified through active case detection in six districts of Nepal. medRxiv.

[31] Madarasingha N., Senaviratne J. (2011). A study of household contacts of children with leprosy. Ceylon Med. J..

[32] Gupte M., Murthy B., Mahmood K., Meeralakshmi S., Nagaraju B., Prabhakaran R. (2004). Application of lot quality assurance sampling for leprosy elimination monitoring--examination of some critical factors. Int. J. Epidemiol..

[33] Dharmawan Y., Fuady A., Korfage I., Richardus J.H. (2021). Individual and community factors determining delayed leprosy case detection: A systematic review. PLoS Negl. Trop. Dis..

[34] Henry M., GalAn N., Teasdale K., Prado R., Amar H., Rays M.S., Roberts L., Siqueira P., De Wildt G., Virmond M. (2016). Factors Contributing to the Delay in Diagnosis and Continued Transmission of Leprosy in Brazil—An Explorative, Quantitative, Questionnaire Based Study. PLoS Negl. Trop. Dis..

[35] Dharmawan Y., Fuady A., Korfage I.J., Richardus J.H. (2022). Delayed detection of leprosy cases: A systematic review of healthcare-related factors. PLoS Negl. Trop. Dis..

[36] Schoenmakers A., Hambridge T., van Wijk R., Kasang C., Richardus J.H., Bobosha K., Mitano F., E Mshana S., Mamo E., Marega A. (2021). PEP4LEP study protocol: Integrated skin screening and SDR-PEP administration for leprosy prevention: Comparing the effectiveness and feasibility of a community-based intervention to a health centre-based intervention in Ethiopia, Mozambique and Tanzania. BMJ Open.

[37] Mohanty P.S., Bansal A.K., Naaz F., Patil S.A., Arora M., Singh M. (2020). Dominant marker (inter-simple sequence repeat-polymerase chain reaction) versus codominant marker (RLEP-polymerase chain reaction) for laboratory diagnosis of leprosy: A comparative evaluation. Int. J. Mycobacteriol..

