# Supplementary material for: Exploring Active Case Detection Approaches for Leprosy Diagnosis in Varied Endemic Settings: A Comprehensive Scoping Review
Source: Life (Basel). 2024 Jul 26;14(8):937. doi: 10.3390/life14080937 (PMC11355679; doi:10.3390/life14080937)
Supplement: Supplementary file 1 [file life-14-00937-s001.zip › S1_Figure_I-SAM Model.pdf]

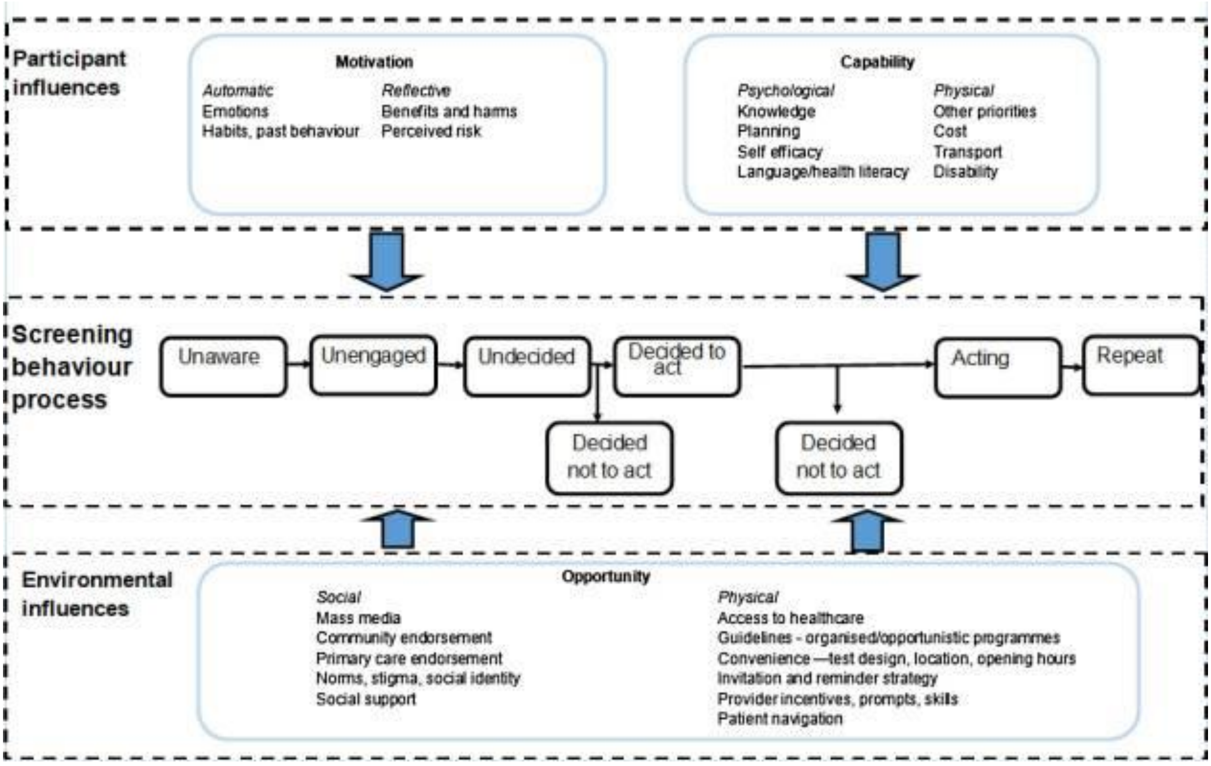

I-SAM model, a theory-based approach for screening program development and implementation (Reprinted from Ref. [7]).
