# Supplementary material for: Exploring Active Case Detection Approaches for Leprosy Diagnosis in Varied Endemic Settings: A Comprehensive Scoping Review
Source: Life (Basel). 2024 Jul 26;14(8):937. doi: 10.3390/life14080937 (PMC11355679; doi:10.3390/life14080937)
Supplement: Supplementary file 1 [file life-14-00937-s001.zip › S2_Search String Table.pdf]

Search strings used for the three databases searched.

Database	Search String	Number of Results		Filters applied	New Number of Results
PubMed	("leprosy"[MeSH Terms] OR "mycobacterium leprae"[MeSH Terms] OR "Multibacillary"[Title/Abstract] OR "paucibacillary"[Title/Abstract] OR "leprae"[Title/Abstract] OR "hansen disease"[Title/Abstract] OR "leprosy"[Title/Abstract]) AND ("diagnosis"[MeSH Terms] OR "Isolation and Purification"[MeSH Subheading] OR "Contact tracing"[MeSH Terms] OR "Detection"[Title/Abstract] OR "screen*"[Title/Abstract] OR "case find*"[Title/Abstract] OR "case detect*"[Title/Abstract] OR "contact trac*"[Title/Abstract] OR "contact investig*"[Title/Abstract] OR "diagnosis"[Title/Abstract] OR "Isolation and Purification"[Title/Abstract] OR "Contact tracing"[Title/Abstract]) AND ("epidemiology"[MeSH Terms] OR "prevalence"[MeSH Terms] OR "incidence"[MeSH Terms] OR "Endemic"[Title/Abstract] OR "area*"[Title/Abstract] OR "prevalen*"[Title/Abstract] OR "incidence"[Title/Abstract])	2,372		Years 2000 to 2023 English Language	**1,545**
Embase	(exp leprosy/ or (Leprosy or leprae or Multibacillary or paucibacillary or "hansen disease").ti,ab,kf.) AND (exp diagnosis/ or exp "isolation and purification"/ or exp contact examination/ or ("Detection" or "screen*" or "case find*" or "case detect*" or "contact trac*" or "contact investig*" or "diagnosis" or "Isolation and Purification" or "Contact tracing").ti,ab,kf.) AND (exp epidemiology/ or exp prevalence/ or exp incidence/ or ("Endemic" or "area*" or "prevalen*" or "incidence").ti,ab,kf.)	4,161		Years 2000 to 2023 English Language	**2,925**
Web of Science	(TS=(Leprosy or leprae or Multibacillary or paucibacillary or "hansen disease")) AND (TS=("Detection" or "screen*" or "case find*" or "case detect*" or "contact trac*" or "contact investig*" or "diagnosis" or "Isolation and Purification" or "Contact tracing")) AND (TS=(epidemiology OR "Endemic" or "area*" or "prevalen*" or "incidence"))	1,522		Years 2000 to 2023 English Language	**1,271**
