# Supplementary material for: Exploring Active Case Detection Approaches for Leprosy Diagnosis in Varied Endemic Settings: A Comprehensive Scoping Review
Source: Life (Basel). 2024 Jul 26;14(8):937. doi: 10.3390/life14080937 (PMC11355679; doi:10.3390/life14080937)
Supplement: Supplementary file 1 [file life-14-00937-s001.zip › S3_Data Extraction Table.pdf]

Data Extraction Table

Reference	Author(s)	Title	Publication Year	Location	Country	Case detection Method	Diagnostic Technique Used	Endemicity	Setting	Screening Program Sample Size	Results (Effectiveness)
[9]	F. Bernardes Filho, C. M. L. Silva, G. Voltan, M. N. Leite, A. Rezende, N. A. de Paula, et al.	Active search strategies, clinicoimmunobiological determinants and training for implementation research confirm hidden endemic leprosy in inner São Paulo, Brazil	2021	Jardinopolis, São Paulo, Brazil	Brazil	Screening Questionnaire Distribution	Physical Medical Examination (PME)<br><br>Anti- anti-phenolic glycolipid-I (PGL-I) serology<br><br>Polymerase Chain Reaction (PCR)	Low	Community	3,241 Leprosy Screening Questionnaires (LSQs) were issued	64 Diagnosed
[10]	M. L. Moura, K. M. Dupnik, G. A. Sampaio, P. F. Nóbrega, A. K. Jeronimo, J. M. do Nascimento-Filho, et al.	Active Surveillance of Hansen's Disease (Leprosy): Importance for Case Finding among Extradomiciliary Contacts	2013	The municipality of Mossoro, Rio Grande do Norte, Brazil	Brazil	Household contact/Social contact (HHC/SC) Identification and Screening<br><br>Door-to-Door Screening	PME<br><br>Semmes-Weinstein monofilaments used for light touch sensation testing<br>Referred to a specialist for further investigation	High	Community	719 Screened	15 people diagnosed

[11]	J. A. C. Nery, M. I. F. Pimentel, M. R. Lyra, B. L. von Sohsten, D. P. Marinho and A. R. S. Perisse	Detection of clusters of leprosy cases among Guarani Indians in the Southern Region of the state of Rio de Janeiro, Brazil	2012	Indigenous population living in communities in the State of Rio de Janeiro, Brazil	Brazil	Door-to-Door Screening	PME	High	Community	255 Screened	21 Cases were identified
[12]	C. M. Lincoln Silva, F. B. Filho, G. Voltan, J. M. Santana, M. N. Leite, F. R. Lima, et al.	Innovative tracking, active search and follow-up strategies for new leprosy cases in the female prison population	2021	Ribeirão<br><br>Preto Female Penitentiary, Brazil	Brazil	Screening Questionnaire Distribution	PME<br><br>Peripheral nerve ultrasonography Anti-PGL-I serology.	High	Prison Population	404 Screened	14 new cases were diagnosed
[13]	D. C. Campos, A. P. Dutra, V. L. Suares, P. A. Carvalho and L. M. Camargo	New strategies for active finding of leprosy cases in the Amazonian region	2015	Municipality of Monte Negro, State of Rondonia, Brazil	Brazil	Screening Questionnaire Distribution<br>HHC/SC Identification and Screening<br><br>Door-to-Door Screening	PME<br><br>Dermal scraping<br>Semmes–Weinstein monofilaments used for light touch sensation testing<br>Reaction to 1% histamine and 1% pilocarpine testing	High	Community	181 Screened	7 new cases diagnosed
[14]	C. M. Sato, T. Rodrigues, P. R. S. Silva, E. S. Dos Santos, D. R. Xavier, I. Baptista, et al.	Social school contacts of multibacillary leprosy cases in children living in the hyperendemic region of the Midwest of Brazil	2022	Cuiaba, Brazil	Brazil	HHC/SC Identification and Screening	PCR Testing	High	School Population	236 Screened	33 new cases were detected

[15]	M. A. Frade, N. A. de Paula, C. M. Gomes, S. Vernal, F. Bernardes Filho, H. B. Lugão, et al.	Unexpectedly high leprosy seroprevalence detected using a random surveillance strategy in midwestern Brazil: A comparison of ELISA and a rapid diagnostic test	2017	Brasilia, Brazil	Brazil	Rapid Village Survey	PME<br><br>Anti-PGL-I and anti- Leprosy Infectious Disease Research Institute (IDRI) Diagnostic-1 (LID-1) antibody titres by ELISA	Low	Community	434 individuals were screened	44 were diagnosed with leprosy
[16]	V. P. Shetty, U. H. Thakar, E. D'Souza, S. D. Ghate, S. Arora, R. P. Doshi, et al.	Detection of previously undetected leprosy cases in a defined rural and urban area of Maharashtra, Western India	2009	State of Maharashtra, India	India	Door-to-Door Screening	PME<br><br>Semmes–Weinstein monofilaments used for light touch sensation testing<br>Slit skin smears<br>Biopsies obtained were stained by Trichome modified Fite-Feraco	High	Community	196,694 Screened - RURAL<br><br>600,247 Screened - URBAN	120 Cases found - Rural<br><br>134 Cases found - Urban

[17]	A. Kumar, A. Girdhar, J. K. Chakma and B. K. Girdhar	Detection of previously undetected leprosy cases in Firozabad District (U.P.), India during 2006-2009: a short communication	2013	Firozabad district of Uttar Pradesh, India	India	Screening Questionnaire Distribution<br>Door-to-Door Screening	PME<br>Slit-skin smears	Low	Community	983,075 screened by researchers	744 previously undetected cases found
[18]	M. S. Kumar, S. Padmavathi, M. Shivakumar, U. Charles, M. Appalanaidu, R. Perumal, et al.	Hidden leprosy cases in tribal population groups and how to reach them through a collaborative effort	2015	Nellore district, Andhra Pradesh, India	India	Screening Questionnaire Distribution<br>Door-to-Door Screening	PME	Low	Community	47,574 people interviewed	70 new leprosy cases identified
[19]	G. Norman, G. A. Joseph, P. Udayasuriyan, P. Samuel and M. Venugopal	Leprosy case detection using schoolchildren	2004	Karigiri, Vellore, India	India	School-Based Screening	PME	High	School Population	23,125 children were screened	14 new leprosy cases were diagnosed
[20]	P. V. Rao, R. A. Bhuskade and K. V. Desikan	Modified leprosy elimination campaign (MLEC) for case detection in a remote tribal area in the State of Orissa, India	2000	State of Orissa, India	India	Door-to-Door Screening	PME<br>Slit-Skin Smears	High	Community	1.47 million people in all villages/hamlets - all screened	576 new leprosy cases detected

[21]	V. P. Shetty, S. S. ya, S. Arora and G. D. Capadia	Observations from a 'special selective drive' conducted under National Leprosy Elimination Programme in Karjat taluka and Gadchiroli district of Maharashtra	2009	Karjat taluka and Gadchiroli district of Maharashtra, India	India	Door-to-Door Screening	PME	High	Community	109,581 Screened - Karjat<br><br>894,971 Screened - Gadchiroli	Confirmed new leprosy cases:<br><br>38 - Karjat<br><br>281 - Gadchiroli
[22]	S. Chen, Y. Zheng, M. Zheng and D. Wang	Rapid survey on case detection of leprosy in a low endemic situation, Zhucheng County, Shandong Province, The People's Republic of China	2007	Shandong Province, The People’s Republic of China	China	Rapid Village Survey	PME	Low	Community	1,360 screened	No Leprosy Cases were found
[23]	N. Wang, T. Chu, F. Li, Z. Wang, D. Liu, M. Chen, et al.	The role of an active surveillance strategy of targeting household and neighbourhood contacts related to leprosy cases released from treatment in a low-endemic area of China	2020	21 counties of Shandong province, China	China	HHC/SC Identification and Screening	PME	Low	Hospital	9,742 were screened	13 new leprosy cases detected

							Slit skin smear test and skin biopsy<br>PCR				
[24]	C. Ruth Butlin, P. Nicholls, B. Bowers, S. Singh, K. Alam and E. Quilter	Household contact examinations: Outcome of routine surveillance of cohorts in Bangladesh	2019	Four districts of north-west Bangladesh	Bangladesh	HHC/SC Identification and Screening	PME	Low	Community	37,334 participants screened	526 new household contact cases identified
[25]	A. Cavaliero, S. S. Ay, A. Aerts, S. Lay, V. So, J. Robijn, et al.	Preventing leprosy with retrospective active case finding combined with single-dose rifampicin for contacts in a low endemic setting: results of the Leprosy Post-Exposure Prophylaxis program in Cambodia	2021	31 operational districts in Cambodia	Cambodia	HHC/SC Identification and Screening	PME	Low	Hospital	7,496 were screened	33 new leprosy cases diagnosed
[26]	N. Cardona-Castro, J. C. Beltrán-Alzate and R. Manrique-Hernández	Survey to identify Mycobacterium leprae-infected household contacts of patients from prevalent regions of leprosy in Colombia	2008	Bolívar / Córdoba and Sucre states, Columbia	Columbia	HHC/SC Identification and Screening	PME<br>Lepromin testing<br>Anti-PGL-I serology<br>PCR Testing	High	Community	402 Screened	54 cases were detected
[27]	N. Ortuno-Gutierrez, A. Mzembaba, A. Baco, S. M. Braet, A.	High yield of retrospective active case finding for leprosy in Comoros	2022	The island of Anjouan, Comoros	Comoros	HHC/SC Identification and Screening	PME	High	Community	133 index case households and 32 nearby households were visited	12 new leprosy patients were diagnosed

	Younoussa, Z. Salim, et al.									945 Screened:<br>671 household contacts<br>274 neighbourhood contacts	
[28]	H. Krismawati, A. Oktavian, Y. Maladan and T. Wahyuni	Risk factor for Mycobacterium leprae detection in household contacts with leprosy patients: A study in Papua, East Indonesia	2020	Papua, Indonesia	Indonesia	HHC/SC Identification and Screening	PCR Testing	High	Community	107 Screened	21 household contacts were diagnosed
[29]	M. S. Utap and A. Kiyu	Active case detection of leprosy among indigenous people in Sarawak, East Malaysia	2017	Sarawak, Borneo, East Malaysia	Malaysia	Door-to-Door Screening	PME<br>Slit skin smear<br>Skin biopsy	High	Community	83 screened	6 new cases identified
[30]	R. K. Mahato, U. Ghimire, M. Lamsal, B. Bajracharya, M. Poudel, P. Naapit, et al.	Epidemiology of leprosy identified through active case detection in six districts of Nepal	2022	Nepal	Nepal	HHC/SC Identification and Screening<br>Prison-Based Screening<br>Door-to-Door Screening	PME	High	Community &<br>Prison Population	1.) 26,469 Screened<br>2.) 7,608 Screened<br>3.) 4,428 Screened	1.) 27 Confirmed cases<br>2.) 19 Confirmed Cases<br>3.) 2 Confirmed cases
[31]	N. P. Madarasingha and J. K. Senaviratne	A study of household contacts of children with leprosy	2011	Lady Ridgeway Hospital, Colombo, Sri Lanka	Sri Lanka	HHC/SC Identification and Screening	PME<br>Slit-skin smear	High	Hospital	311 Screened	51 positive contacts were detected<br>of these 25 were new cases<br>33% of the index cases had a positive contact.<br>11% had more than one

											positive contact
